# Intraventricular Haemorrhage Complicated by Hydrocephalus in an Acutely Encephalopathic Preterm Infant

**DOI:** 10.7759/cureus.2193

**Published:** 2018-02-15

**Authors:** Kene Maduemem, Sameen Khalid, Maya Hariharan, Aamer Siddique

**Affiliations:** 1 Pediatrics, Walsall Manor hospital; 2 Paediatrics and Neonatology, University Hospital, Galway

**Keywords:** acute encephalopathy, intraventricular haemorrhage, hydrocephalus

## Abstract

Intraventricular haemorrhage (IVH) is characterized by bleeding of the immature subependymal germinal matrix in preterm infants, but the pathogenesis is multifactorial. IVH and posthaemorrhagic hydrocephalus (PHH) are common causes of neonatal morbidity and mortality among preterm infants. We describe a preterm male infant who was born clinically stillbirth; became moderately severe encephalopathic. He had bilateral IVH (III right and IV left) with consequent PHH. His incredible outcome following a stormy perinatal period appears intriguing. Long-term follow-up is needed to evaluate the severity of deficits as he matures. Whether therapeutic cooling would have made a difference or not is debatable.

## Introduction

Intraventricular haemorrhage (IVH) is a major neurological complication of prematurity. Posthaemorrhagic hydrocephalus is the most common sequela of IVH with grave morbidity and mortality. The cause of IVH is multifactorial, comprising inherent fragility of germinal matrix vasculature, distortion in cerebral blood flow and platelet and coagulation dysregulations. Hypoxic ischaemic insult is a major contributing factor to disturbance in cerebral perfusion and germinal matrix bleed. Therapeutic hypothermia is the standard treatment option for neonates of gestational age 36 weeks and above with moderate to severe hypoxic ischaemic encephalopathy (HIE). The care of preterms with HIE still remains an area of uncertainty for the neonatal team. We describe the stormy perinatal period of a preterm (33+4 weeks gestation) neonate delivered clinically stillbirth with an incredible outcome.

## Case presentation

**Figure 1:**
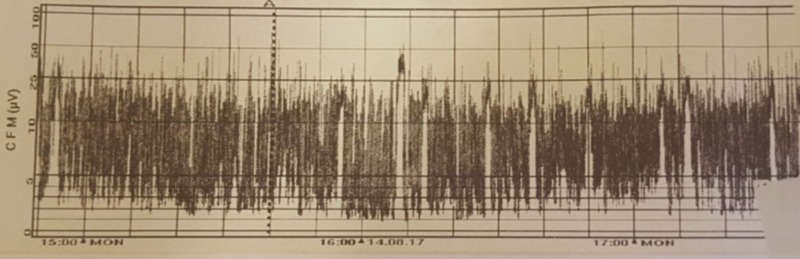
Bedside electroencephalograph (EEG).

A male preterm was born at 33+4 weeks gestation to a 36-year-old Irish mother. Antenatal period had no adverse events. Foetal anomaly scans were normal. Mother’s blood group is O Rhesus negative. Serology tests were negative with rubella immunity. She presented three days prior to delivery with a lower back pain which was thought to be musculoskeletal. She subsequently presented on the day of delivery with a 12-hour history of reduced foetal movement and persisting back pain. A pathological trace was revealed on cardiotocography which warranted an emergency caesarean section. This preterm male neonate was delivered with no sign of life. Placental abruption with a large retroplacental clot was seen. This is the second child of non-consanguineous Irish parents. The first child was born at term by emergency caesarean section because of cord prolapse. Apgar scores were 0, 3 and 3 in the 1st, 5th and 10th minute, respectively. The baby was clinically stillbirth on delivery with a weight of 2376 g. He was immediately intubated, and adrenaline was given endotracheally. Chest compression and intermittent positive pressure ventilation were instituted. Heart rate was heard at the 4th minute of life. Cardiopulmonary resuscitation continued till the 11th minute when heart rate became above 100 beats per minute. Clinical seizures—episodic jerking of the right upper and lower limbs—were noticed on the third day of life. Clinical examination revealed a non-dysmorphic, normothermic but extremely jittery infant with global hypertonia worse on the right than left sides. There was no posturing but eye deviation to the right was noted. Primitive reflexes were depressed. Pupils were normal sized but responded sluggishly to the light. Chest and abdominal examinations were normal. Heart rate and blood pressure were within normal limits. Pre- and post-ductal saturation difference was <2%. A clinical assessment of hypoxic ischaemic encephalopathy (HIE) Sarnat stage II-III was made. With no sign of life, he was immediately resuscitated by administering 0.5 ml of adrenaline endotracheally. Emergency umbilical arterial and venous lines were inserted. He was given 10 ml/kg of 0.9% saline. Blood sugar was 1.7 mmol/L and corrected with 2 ml/kg 10% dextrose bolus. Surfactant (Curosurf) 200 mg/kg was administered. 18 mmol of sodium bicarbonate was calculated as deficit and 9 mmol/L given as bolus. An eligibility criterion for therapeutic cooling was not met because of gestation less than 35 weeks. He was ventilated for 12 hours before weaning to biphasic positive airway pressure (BiPAP). After 18 hours on BiPAP, non-invasive ventilation was switched to high flow oxygen by nasal prongs from days 2 to 6 of life. Clinical seizures observed on the third day of life were aborted with loading dose intravenous phenobarbitone 20 mg/kg. Bedside electroencephalograph (EEG) demonstrated moderately severe encephalopathic activity (Figure 1).

Maintenance dose of 5 mg/kg/day was continued till discharge. Intravenous antibiotics were given for 48 hours and discontinued following negative culture according to local guidelines.

Venous cord gas showed severe metabolic acidosis (pH 6.9, base excess -24.3 mmol/L, lactate 14.3 mmol/L). Acidosis improved following normal saline boluses (arterial blood gas: pH 7.22, base excess -13.9, lactate 4.5). Results of investigations shortly after birth are shown in Table 1.

**Table 1:** Blood test results shortly after birth.

Parameters	Results	Reference range
White blood cell	13.4 × 10^9^/L	10-26
Neutrophil	4.7 ×​​​​​​​ 10^9^/L	4-14
Lymphocyte	7.5 ×​​​​​​​ 10^9^/L	3-8
Monocyte	1.1 ×​​​​​​​ 10^9^/L	0.5-2
Haemoglobin	18.9 g/dL	14-22
Haematocrit	0.567 L/L	0.45-0.75
Nucleated red cells	46/100 White blood cells	
Platelet	226 ×​​​​​​​ 10^9^/L	150-450
Creatine kinase	1643 U/L	39-308
Troponin T	207 mg/dL	0-14
Lactate dehydrogenase	3562 U/L	
Alanine transaminase	124 U/L	0-40
Alkaline phosphatase	206 U/L	40-250
Gamma-glutamyl transferase	114 U/L	10-71
Total protein	43 g/L	64-83
Albumin	32 g/L	39-51
Total bilirubin	89 umol/L	1-100

**Figure 2:**
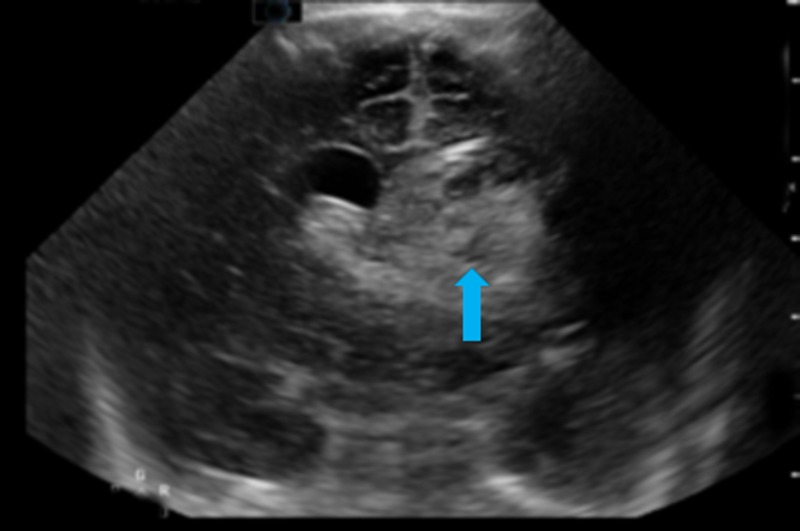
Grade IV left-sided intraventricular haemorrhage (arrow). Grade III right-sided intraventricular haemorrhage. Mild midline shift to the right.

The inflammatory markers were normal. There was negative panculture. Renal function on the second day of life was unremarkable. Coagulation profile, including extended clotting screen, was normal. Figure 2 shows cranial ultrasound scan on the third day of life revealing bilateral IVH.

**Figure 3:**
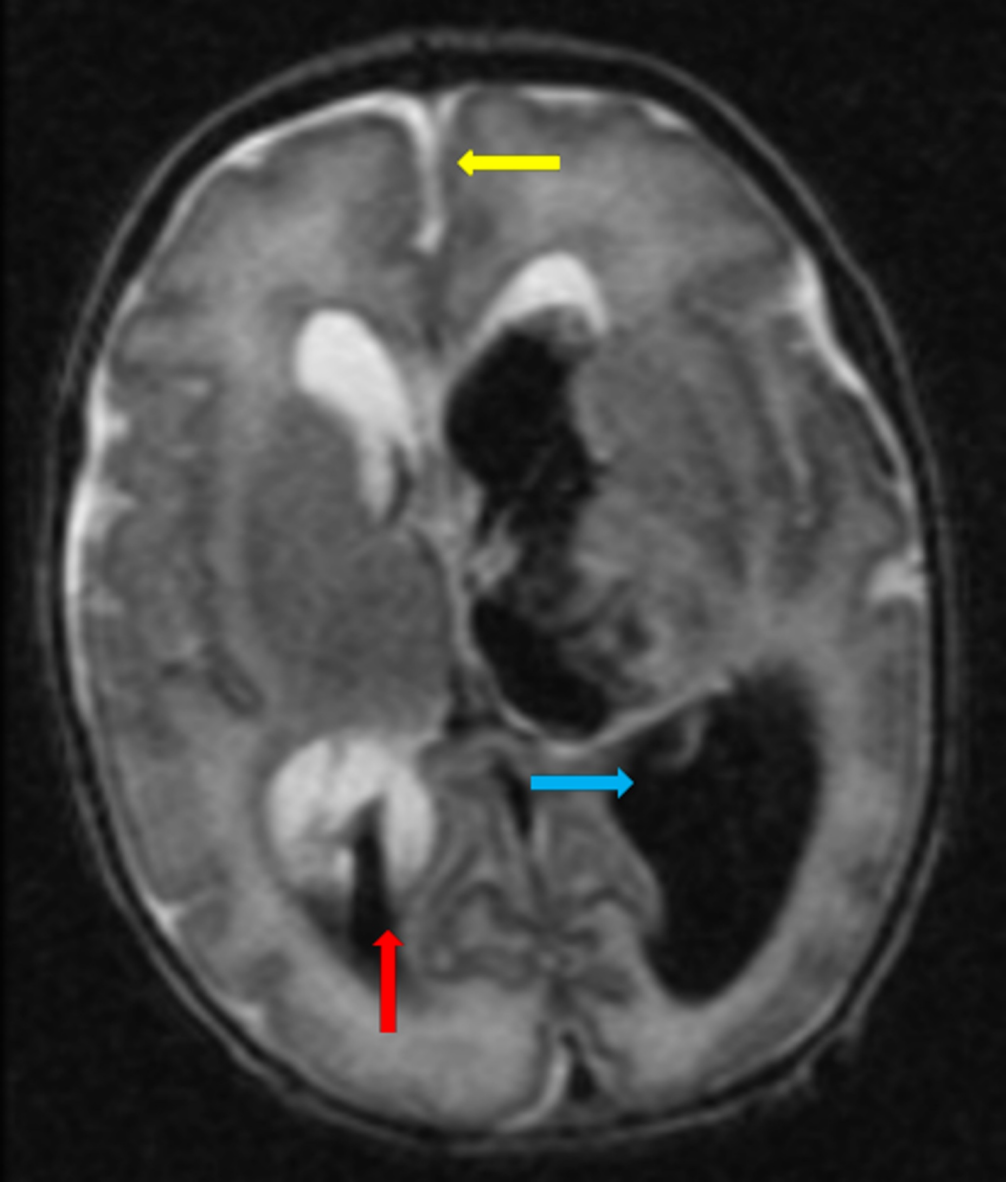
Grade IV left-sided interventricular haemorrhage (blue arrow). Grade III right-sided intraventricular haemorrhage (red arrow). Midline shift is noted (yellow arrow). There is parenchymal haemorrhage extending into the left frontoparietal region.

Brain magnetic resonance imaging on the sixth day of life confirmed left Grade IV and right Grade III IVH with midline shift in Figure 3.

**Figure 4:**
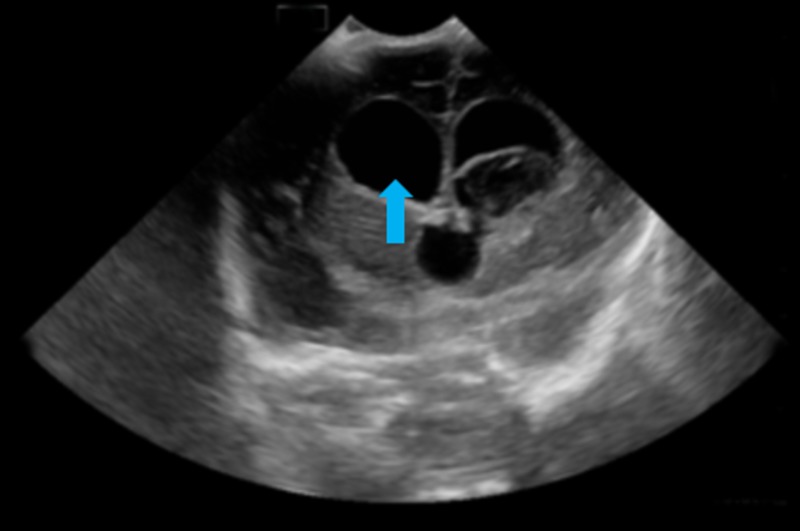
No new haemorrhage. There is marked increase in hydrocephalus involving both lateral ventricles (arrow) and the third ventricle.

Head circumference was noticed to have increased from 50th to 75th centile on the 17th day of life. There was splaying of cranial sutures and bulging anterior fontanelle. Cranial ultrasound scan in Figure 4 revealed post-haemorrhagic hydrocephalus.

This subsequently plateaued both clinically and radiologically. Neurosurgical consult was sought in a paediatric tertiary centre given the increase in head circumference. He was managed conservatively because of stable neurological and haemodynamic states till discharge. He was fully bottle fed on discharge at 40+1 weeks postconceptual age. The phenobarbitone therapy was continued with the view of weaning off as he grows. Multidisciplinary team follow-up is ongoing. Parents understood that prognosis is guarded but long-term follow-up will reveal any neurological deficits.

## Discussion

IVH is defined as the bleeding within the germinal matrix due to rupture of fragile blood vessels in the developing brain. IVH is graded from I to IV based on extent and severity [1]. IVH occurs in infants of 32 weeks’ gestation with inverse relationship between IVH and gestational age. The pathogenesis of IVH is multifactorial; involving an interplay of three factors: i) fragility of germinal matrix vasculature, ii) distortion of cerebral blood flow and iii) platelet and coagulation dysregulations. Certain predisposing factors have been shown to lead to IVH but not limited to low Apgar score, respiratory distress syndrome, hypoxia and hypercapnia, asphyxia, sepsis. Our patient suffered significant hypoxic-ischaemic insult secondary to a placental abruption. We hypothesized that the degree of resuscitation may have been a contributory factor to IVH development probably due to blood pressure fluctuations.

Placental abruption is the premature separation of the placenta before delivery. The bleeding can be revealed or concealed; the latter was the hallmark in the index case. There are several risk factors associated with placental abruption, but the pathogenesis is not well understood. Atkinson, et al. demonstrated in their study, the risk factors and perinatal outcome of placental abruption [2].

HIE in term babies is diagnosed with well-defined neurological and biochemical parameters. Therapeutic hypothermia has been extensively studied and deemed safe in neonates of gestational age 36 weeks and above [3-5]. Preterms below 35 weeks gestation were excluded in those clinical trials because of insufficient evidence for efficacy and safety. There are varying criteria in defining HIE in preterms. Gopagondanahalli, et al. proposed definitions of HIE in preterm neonates. Two criteria would be met for a definite HIE; i) pH ≤ 7 and base deficit ≥ 12 mmol/L in fetal/cord/first neonatal blood sample, ii) neonatal encephalopathy – Sarnat staging (staged according to all criteria for infants between 33 and 35 weeks of gestation), significant changes in neurological examination and/or seizures (for infants less than 33 weeks of gestation) [6]. The index case met both criteria but did not qualify for therapeutic cooling because of gestational age.

Post-hemorrhagic hydrocephalus (PHH) is a significant sequela of IVH. It occurs in 30-50% of all preterm infants with a grade III or IV IVH, and 25-30% of these develop progressive hydrocephalus [7]. PHH can be communicating or obstructive (non-communicating). Cerebrospinal fluid reabsorption can be impaired if thrombi are obstructing the arachnoid villi or granulations. Patients with PHH usually present clinically with increasing head circumferences, signs of increased intracranial pressure. However, clinical manifestations of hydrocephalus may not be evident for several weeks post-hemorrhage due to the compliance of neonatal brain. Controversies exist regarding the timing and mode of intervention [8]. Intervention may be medical or surgical. Our patient was managed conservatively owing to the plateau of the head circumference. Any intervention will depend on the subsequent follow-up by the neurosurgical team.

Perinatal HIE is a major cause of neurodevelopmental impairment. The risk of cerebral palsy has been well documented in the literature. The cause of cerebral palsy is multifactorial, with prematurity having the greatest impact as demonstrated by Sukhov, et al. [9]. Asphyxia and adverse labour events also accounted significantly to the development of cerebral palsy. Thorngren-Jerneck and Herbst in a Swedish study showed prematurity and placental abruption as significant perinatal factors associated with cerebral palsy [10]. Severe IVH represents a significant risk factor for poor neurodevelopmental outcome. The extent of neurological deficits in our patient is largely unknown. Follow-up will unravel any form and severity of neurological sequelae.

## Conclusions

Radiological findings in IVH do not always correlate with clinical findings. Considering therapeutic hypothermia in preterms with IVH continues to pose a huge challenge. Discussing the prognosis of an infant with a stormy perinatal period with parents is overwhelmingly tricky as time is probably the major determining factor.

## References

[1] Papile LA, Burstein J, Burstein R (1978). Incidence and evolution of subependymal and intraventricular haemorrhage: a study of infants with birth weights less than 1,500 gm. J Pediatr.

[2] Atkinson AL, Santolaya-Forgas J, Blitzer DN (2015). Risk factors for perinatal mortality in patients admitted to the hospital with the diagnosis of placental abruption. J Matern Fetal Neonatal Med.

[3] Shankaran S, Laptook AR, Ehrenkranz RA (2005). Whole body hypothermia for neonates with hypoxic-ischemic encephalopathy. N Engl J Med.

[4] Simbruner G, Mittal RA, Rohlmann F (2010). Systemic hypothermia after neonatal encephalopathy: outcomes of neo.nEURO.network RCT. Pediatrics.

[5] Jacobs SE, Morley CJ, Inder TE (2011). Whole body hypothermia for term and near-term newborns with hypoxic-ischemic encephalopathy: a randomized controlled trial. Obstet Gynecol Surv.

[6] Gopagondanahalli KR, Li J, Fahey MC (2016). Preterm hypoxic–ischemic encephalopathy. Front Pediatr.

[7] Murphy BP, Inder TE, Rooks V (2002). Post-haemorrhagic ventricular dilatation in the premature infant: natural history and predictors of outcome. Arch Dis Child Fetal Neonatal Ed.

[8] de Vries LS, Liem KD, van Dijk K (2002). Early versus late treatment of posthaemorrhagic ventricular dilatation: results of a retrospective study from five neonatal intensive care units in The Netherlands. Acta Paediatr.

[9] Sukhov A, Wu Y, Xing G (2012). Risk factors associated with cerebral palsy in preterm infants. J Matern Fetal Neonatal Med.

[10] Thorngren-Jerneck K, Herbst A (2006). Perinatal factors associated with cerebral palsy in children born in Sweden. Obstet Gynecol.

